# Conventional and innovative approaches to black fungi control for stone heritage preservation

**DOI:** 10.1002/iub.70010

**Published:** 2025-03-20

**Authors:** Domenico Celi, Massimiliano Marvasi, Brunella Perito

**Affiliations:** ^1^ Department of Biology University of Florence Sesto Fiorentino Italy

**Keywords:** antimicrobial treatment, Black Meristematic Fungi, conventional and innovative biocides, ecological transition, rock inhabiting fungi, stone monuments biodeterioration, stress resistance

## Abstract

Black Meristematic Fungi (BMF) are characterized by a thick melanized cell wall and an isodiametric cellular expansion. BMF represent one of the most damaging groups of microorganisms causing the deterioration of outdoor exposed stone monuments mainly due to the formation of dark spots and patches leading to the darkening of their surface, cracking, and bio‐pitting. BMF are among the most stress‐resistant organisms on Earth, known for their remarkable ability to withstand solar radiation, desiccation, and extreme temperature fluctuations, which has led to their widespread distribution across the globe. These features make BMF very difficult to remove and restrict, representing a challenge for restorers. Despite the number of scientific works about BMF isolation and ecology, little is known about their response to antimicrobial treatments. Conventional biocides remain the most used treatment for the control of biodeterioration on stone artworks. In recent years, interest in alternative and safer antimicrobial treatments has risen in conservation strategies. The number of scientific works in which their efficacy against BMF is evaluated is, however, still low. The aim of this review is to assess existing studies regarding the response of BMF to both conventional and innovative treatments. This will encompass an in‐depth examination of methodologies for the application and evaluation of treatments. Furthermore, we aim to highlight future research directions that will contribute to a more informed selection of effective anti‐BMF interventions for stone preservation. We underscore the significance of pioneering, environmentally low‐impact solutions.

## 1 INTRODUCTION

**FIGURE 1:**
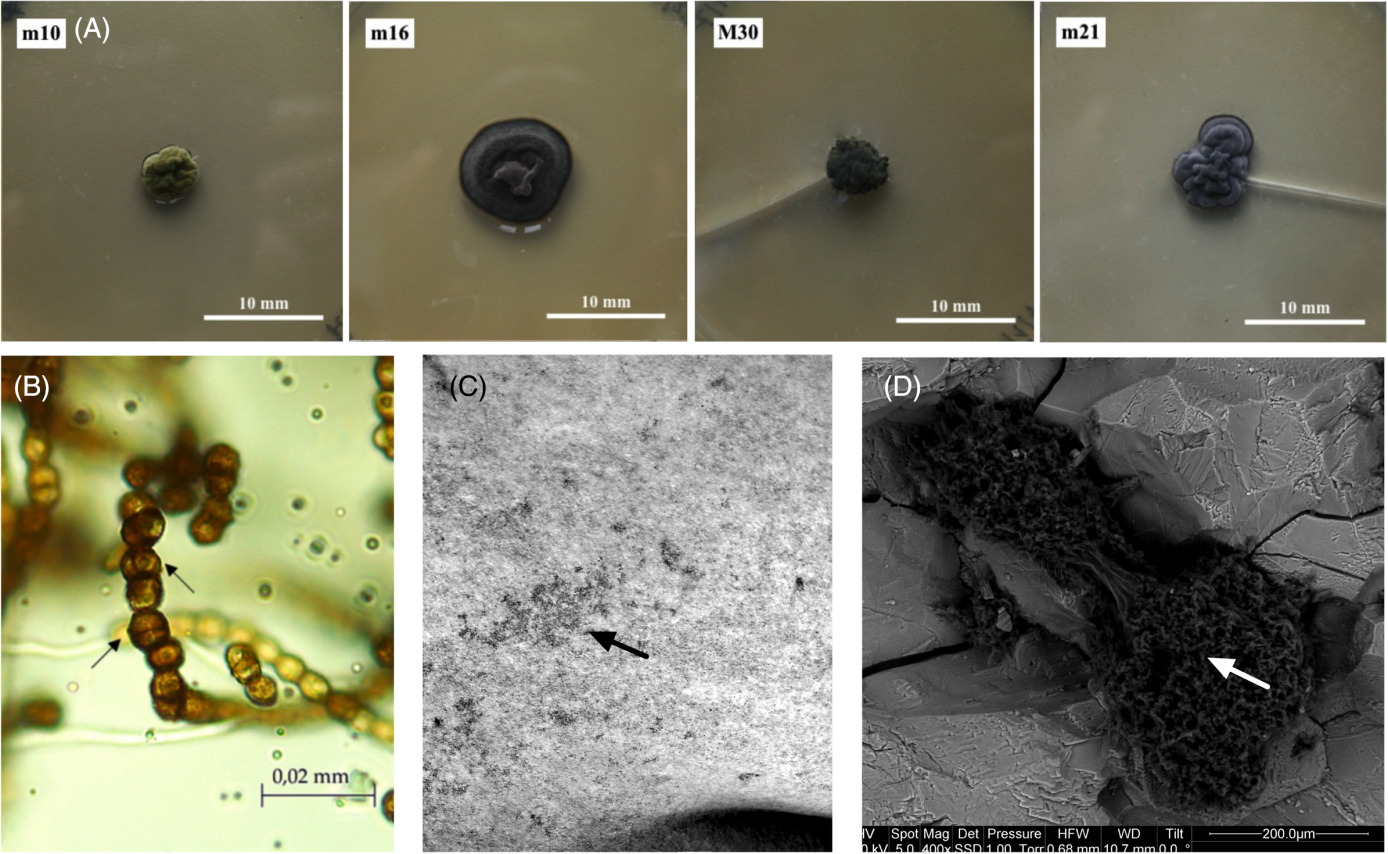
Black Meristematic Fungi (BMF) on stone. (A) Phenotypic variety of colonies of four BMF strains isolated from the darkened marble of Florence Cathedral: M10, *Vermiconia* sp.; m16, *Knufia* sp.; M30, *Coniosporium* sp.; m21, *Lithohypha* sp.^6^; (B) Meristematic cells of the newly identified fungus *Saxispiralis lemnorum*, isolated from deteriorated limestone, subdivided by septations in various directions (arrows).^7^ (C) Close‐up of the ‘Ratto delle Sabine’ marble sculpture in Florence, Italy, revealing colonization by *Knufia petricola* (syn. *Sarcinomyces petricola*) as darkened patches; (D) Scanning Electron Microscope image highlighting a *K. petricola* colony isolated from the ‘Ratto delle Sabine’ within a marble microcrack, arrow indicating the main growth area.

Black Meristematic Fungi (BMF) are a morphoecological category of microorganisms characterized by basic traits such as a thick cell wall, the ability to reproduce by unicellular growth, deep melanization, meristematic growth, and usually a very slow growth rate.^1^, ^2^, ^3^, ^4^, ^5^ The term “meristematic fungi” was first used by de Hoog in 1977 to describe fungi characterized by aggregates of thick‐walled, melanized cells enlarging and reproducing by isodiametrical division,^5^ (Figure 1A,B). Black yeasts (BY) are a group of fungi characterized by a melanized cell wall that forms daughter cells by yeast‐like multilateral or polar budding.^2^, ^5^ BMF and BY are simply called Black Fungi (BF), and they include phylogenetically heterogeneous taxa belonging to the classes Dothideomycetes, Eurotiomycetes, and Arthoniomycetes. Some BF lineages have adapted to thrive on rock surfaces and are recognized as natural residents of rocky environments. They are known as Rock Inhabiting Fungi (RIF), highlighting their integral role in the ecosystems of rock surfaces.^1^, ^2^, ^3^, ^4^ Different terms have been used to name these fungi and describe their ecological habits and characteristics. Recently, Liu and collaborators extensively reviewed RIF, providing a nomenclature to refer to these fungi unambiguously.^2^ BMF and BY are polyextremotolerant microorganisms because of their ability to thrive in many extreme environmental conditions such as high solar radiation, temperature fluctuations, osmotic stress, extreme oligotrophic conditions, etc.^8^, ^9^, ^10^, ^11^, ^12^ Their ability to resist such stresses leads them to colonize rocks in extreme environments like hot^13^ and dry deserts^14^; hypersaline waters in salterns,^11^ natural and monumental stones particularly in the Mediterranean Area,^15^, ^16^ but also unusual environments like household refrigerators, as in the case of the BY *Aureobasidium subglaciale*.^17^ In some studies, the RIF resistance mechanisms to hostile factors have been elucidated; nevertheless, they remain generally poorly understood.^18^ According to Tesei,^8^ the stress tolerance mechanisms of BMF can be divided into morpho‐physiological traits and adaptation at the molecular level. Morpho‐physiological traits consist of strategies like phenotypic plasticity, high melanization, simple life cycle (and asexuality), oligotrophism, and accumulation of osmo‐ and cryoprotectants.^8^ Phenotypic plasticity is observed in many species, and based on the habitat conditions, a filamentous to yeast (and reverse) growth switch can be observed. The RIF microcolonial growth, consisting of clump‐like colonies of heavily melanized cells surrounded by thick walls, helps to contrast desiccation and heat shocks by optimizing the surface volume ratio.^8^ In the extreme halotolerant *Hortaea werneckii*, one of the principal responses to high extracellular salinity was demonstrated to be the accumulation of glycerol as a compatible solute up to the maximal concentration of 2.94 mmol/g of dry weight during exponential growth in 25% NaCl.^19^ Exposure to high salinity also leads to an immediate response in cellular dehydration serving as a signal for the activation of the high osmolarity glycerol pathway in yeasts,^20^ some elements of which were found in *H. werneckii*.^21^ On the other hand, our understanding of the adaptations at the molecular levels for resistance and tolerance mechanisms in BMF and BY has significantly advanced with the development of molecular technologies for studying genomics, transcriptomics, and proteomics.^8^ Genetic studies based on sequencing and karyotyping were hampered in the past because of the thick melanized cell walls of these fungi resulting in failed DNA extraction.^22^ Today we are still able to perform genome sequencing only on a few of the known species of BMF/BY, due to this complex cell wall.^8^ Information from genomic studies carried out on the RIF *Cryomyces antarcticus* (genome size: 24 Mbp) indicates the absence of differences at the level of genome size and features (such as genomic duplication or shrinkage) compared with comparative species and mesophilic hyphomycetes, while in the Antarctic species *Friedmanniomyces endolithicus*, a large genome size (46.75 Mbp) accompanied by multiple genes devoted to stress resistance (oxidative stress, UV irradiation, DNA damage, and salt stress) as well as genomic traits unique to this species associated with meristematic growth and cold resistance was observed. Proteomic and transcriptomic analyses of the RIF revealed a general downregulation of metabolism in response to stress, consistent with genomic data.^8^

The stress resistance of BMF could also have potential implications in different fields, from agriculture to medicine.^23^ Furthermore, understanding the stress resistance mechanisms that characterize BMF is crucial for developing more effective strategies to control their growth on monumental stones. In fact, BMF represent one of the most damaging groups of microorganisms causing deterioration of outdoor exposed stone monuments^24^, ^25^ mainly due to the formation of dark spots and patches leading to the darkening^6^, ^26^ of the stone surface (Figure 1C) and penetration into the stone using already existing (micro)fractures (Figure 1D) and causing cracking and bio‐pitting.^6^, ^27^, ^28^ This ability of BMF can be attributed not only to mechanical stress by hyphae, but also to the acid production that can dissolve the stone itself.^6^ In some cases, the penetration into the stone is active, as for *Knufia petricola*, able to slowly penetrate into freshly exposed stone probes thanks to the production of siderophore‐like iron‐chelating molecules.^29^, ^30^, ^31^ Because of their high damaging power as well as their adaptation and resistance to environmental stress, BMF represent a hard challenge for conservators and restorers. In addition to the high difficulty in removing them from the stone, BMF result to be one of the first colonizers after the cleaning procedures.^29^, ^32^ Despite their recognized role in stone biodeterioration, little is known about their response to the treatments used for their containment.

Most of the available works to contrast biodeterioration are based on the use of conventional large spectrum biocides which still are the most used and efficient methods for the control of microbial communities on stone artworks. They result, however, to be potentially dangerous for the operator, the environment, and the artwork itself, enhancing the bio‐receptivity of the stone.^33^, ^34^ In recent years, the search for more sustainable strategies has been developing, and new cleaning procedures using less harmful molecules have been tested in cultural heritage (CH) conservation.^35^ Promising safer alternatives to conventional biocides are represented by nanoparticles (NPs),^36^ ionic liquids with antifouling properties,^37^ and biocides of natural sources.^38^

The aim of this review is to offer an overview of the methods currently tested to contrast the growth of BMF and BY on stone heritage and of the present knowledge on the sensitivity (or resistance) of these fungi to the treatments (Sections 2, 4). To do this, a comprehensive literature review was conducted to explore the treatment of rock‐inhabiting fungi and meristematic black fungi using antimicrobial treatments. The search strategy included four academic databases: Pubmed, Google Scholar, Web of Science, and Scopus. The search queries used were “Rock inhabiting fungi AND biocide treatment” and “Meristematic black fungi AND biocide treatment,” applied consistently across all databases to ensure comparability. The results yielded a number of relevant studies: for “Rock inhabiting fungi AND biocide treatment,” three articles were found in Pubmed, 26 in Google Scholar, five in Web of Science, and two in Scopus; for “Meristematic black fungi AND biocide treatment,” there were two articles in Pubmed, one in Google Scholar, four in Web of Science, and four in Scopus. Articles were screened based on antimicrobial treatments of RIF in laboratory and/or in field conditions. Data were extracted on fungal species, biocides used, and treatment outcomes, contributing to a systematic understanding of this subject. Nine papers were specifically selected from the databases for the core analysis, while the rest were utilized for the introduction and discussion sections. Moreover, a further work based on an antimicrobial physical method was also considered. The information on chemical assays on BMF used for this review is summarized in Table 1.

**TABLE 1:** Literature‐derived anti‐RIF chemical treatments for heritage stone conservation.

RIF tested	Isolated from	Antimicrobial treatment	Treatment evaluation	Results	References
18 strains belonging to: *Aureobasidium*, *Coniosporium*, *Exophiala*, *Knufia*., *Neodevriesia*, *Neophaeotheca*, *Saccotheciaceae*, *Salinomyces*, *Saxophila*, *Vermiconidia*	Carrara marble. Monumental cemetery of Bonaria, Cagliari (Italy)	*In lab*. BZC, LICH, Biotin R and PREV at different concentrations.	ADT, measurement of inhibition halo areas.	Biotin R the most effective biocide.	^18^
Eight strains belonging to *Knufia* sp., *Vermiconia* sp., *Lithohypha* sp., *Coniosporium* sp., *Dothideomycetes* sp.	Carrara marble. Santa Maria del Fiore Cathedral, Florence (Italy)	*In lab*. Origanum and Thyme Essential Oils (EOs), Biotin T, at different low concentrations.	Colony growth (diameter). Statistical analysis.	Both the EOs performed better than the Biotin T at the highest concentration (0.025%).	^6^
*Aureobasidium* MC 875	Unspecified deteriorated stone	*In lab*. 8 different ionic liquids on plate, then on marble and tufa specimens.	ADT, measurement of inhibition halo areas, macroscopic observation, fluorescence microscopy.	2 ILs inhibited the growth of *Aureobasidium* on plate and showed the highest biocidal and preventive activity on marble.	^39^
*Exophiala*, *Cyphellophora*	Macco stone (yellow calcarenite rich in small shells). Monterozzi necropolis, Tarquinia (Italy)	*In lab*. BZC 90%, PREV.	ADT, measurement of inhibition halo areas.	BZC acted better than PREV against *Exophiala* sp. and *Cyphellophora* sp.	^40^
*Coniosporium uncinatum* (CBS100212), *C. apollinis* (CBS109867), *C. perforans* (CBS885.95) *Phaeococcomyces chersonesos* (AJ507323.4)	Travertine (*P. chersonesos*), Roman Theatre of Aosta (Italy); Pentelic marble (*C. apollinis*, *C*. perforans) Sanctuary of Delos (Cyclades, Greece); Carrara marble (*C. uncinatum*), Messina Museum (Italy).	*In lab*. Lichen secondary metabolites: usnic acid, norstictic acid, parietin; BZC.	ADT, measurement of inhibition halo areas	The three metabolites highly effective at 0.01 M in comparison to BZC 1%).	^41^
*Aureobasidium*	Two old buildings of unspecified material.	*In lab*. 6 essential oils tested on plate.	ADT, measurement of inhibition halo diameters.	*T. vulgaris, T. serpyllum* and *F. vulgare* EOs showed the best biocidal activity.	^42^
Strains of the communities analysed before treatment including: *Aureobasidium*, *Cyphellophora*; *Devriesia*	Wall covered in plaster, Villa dei Papiri, Naples (Italy).	In situ. 3 preliminary 5% Biotin R treatments; after 1 month, TiO_2_, and TiO_2_ + Ag nanoparticles	Microscopic analysis, cultivation, molecular identification.	*Cladosporium* still present after treatment while the two BY disappeared.	^43^
*Knufia mediterranea*, *K. marmoricola*	Ordinary and statuary Carrara marble, Monumental Cemetery of Cagliari (Italy).	In situ. DMSO‐based gel (Gel 1); water‐based gel as control (Gel 2); 3% PREV, 2% LICH, 5% Biotin R.	Variation of: Total Color ΔE*, lightness ΔL*, red/green ratio Δa*, yellow/blue ratio Δb*; optical microscopy and macroscopic evaluation.	Gel 1 the best cleaner and biocide	^44^
*Neodevriesia*, *K. petricola*, *K. mediterranea*, *K. marmoricola*, *Exophiala oligosperma*, *Vermiconidia calcicola*, *A. pullulans*, *C. uncinatum*.	Carrara marble, Monumental Cemetery of Cagliari (Italy)^44^	In situ. DMSO‐based gels^45^; Biotin T.	Long‐term evaluation (2, 5, 9 years after treatment). RIF isolation and identification	After 9 years, the Biotin T treatment produced an increase in BF count, biodiversity and resistant species.	^45^

Abbreviations: ADT, agar diffusion test; BZC, benzalkonium chloride; EOs, essential oils; LICH, Lichenicida 264; PREV, Preventol RI 50.

To present the retrieved data, we used a first division in chemical and physical antimicrobial methods. Among chemical methods, we distinguished biocides from chemical synthesis and from natural sources. Despite the great relevance of biodeterioration by RIF, the subject is still in its infancy, as also indicated by the limited number of works available in the literature. Future research directions that will contribute to a more informed selection of effective anti‐BMF interventions for stone preservation are also proposed in Section 5.

## 2 BIOCIDES FROM CHEMICAL SYNTHESIS

In the European Union, biocides are regulated under specific frameworks. According to the Biocidal Products Regulation (BPR), Regulation (EU) 528/2012, biocidal products are substances or mixtures intended to destroy, deter, render harmless, prevent the action of, or otherwise exert a controlling effect on harmful organisms.^35^, ^46^ Biocides can have different applications. The Annex V of the BPR, Regulation (EU) 528/2012, identifies four main groups of biocides, depending on their use. In this review, the term biocides refers to chemicals/formulations that are used to kill and/or remove microorganisms, usually organized to form a biofilm, on the surfaces of stone heritage materials. Biocidal products suitable for preventive or restorative treatments of building materials are classified under product type 2 of main group 1 (disinfectants) and product type 10 of main group 2 (preservatives) in the aforementioned Annex V.^35^ Each biocidal product contains one or several active substances that are designed to control organisms, as reported in the EU reference database (https://echa.europa.eu/home).

### 2.1 Traditional biocides

Although the term “traditional” or “conventional” biocides appears frequently in the literature on cleaning and treatments of stone materials, it is difficult to find a definition. In this review, the term “conventional/traditional biocides” refers to commercial large spectrum biocides widely used in preventive or curative treatments of stone heritage.

Traditional biocides have been widely used for any kind of cultural heritage material for a long time. However, the use of biocides is also increasingly being questioned, as more and more research and publications are demonstrating their toxic effects on human health and the environment,^35^ although they differ in their toxicological profile, as the EU reference database (https://echa.europa.eu/home) indicates.^38^

Moreover, research studies have demonstrated a potential risk of interference with stone materials and an increase in bio‐receptivity of the substrate.^38^ Biocidal treatments have short‐term efficacy and often require frequent reapplication, posing recurring risks to both heritage materials and the environment. In addition, repeated biocidal treatments can cause resistance in target biological agents, damage to nontarget organisms, and favor the growth of more harmful biodeteriogens.^47^ Despite their toxicity and the other side effects, traditional biocides remain the most used practical solution employed to combat biodeterioration.^38^, ^47^

The commercial biocides tested against BMF both in the lab and in the field in the works revised for this review are: Benzalkonium chloride (CTS, Italy), Lichenicida 264 (Bresciani srl, Milano, Italy), Biotin T (CTS, Italy), Biotin R (CTS, Italy), Preventol RI 50 (Bresciani srl, Italy). Their main characteristics (chemical composition and mechanism of action) are described below and synthetically in Table 2.

**TABLE 2:** Products and active molecules employed to control RIF growth in the works considered for this review. In the case of essential oils (EOs), since they are a very complex mixture of chemicals, only the main molecules known to have biocidal action have been reported.

Active molecule(s)	Biocidal product	Structure	Class	Mode of action	CAS number	References
Benzalkonium chloride	Benzalkonium chloride, Preventol RI 50	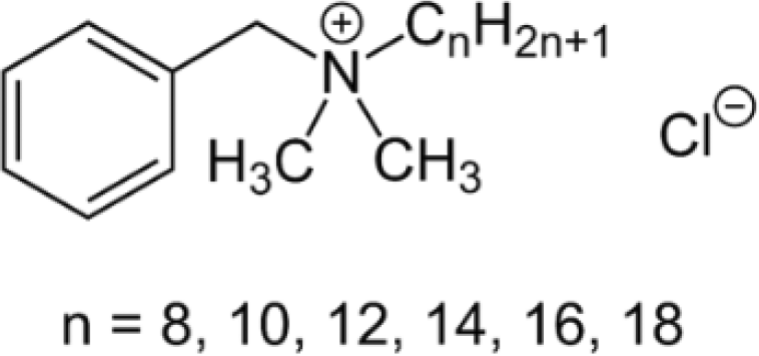	Quaternary Ammonium Compounds (QAC)	Inactivation of energy‐producing enzymes, denaturation of essential proteins, disruption of cellular membrane	63,449‐41‐2	^48^, ^49^
Dodecyldimethylammonium chloride (DDAC)	Biotin T	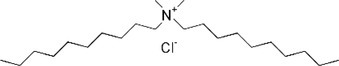			7173‐51‐5	
Formic acid	Biotin T	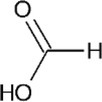	Organic carboxylic acid	Enzyme denaturation and inhibition, cellular structure disruption, impairment of cellular metabolic pathways	64‐18‐6	^50^
2‐N‐octyl‐4‐isothiazolin‐3‐one (OIT)	Biotin R, Biotin T	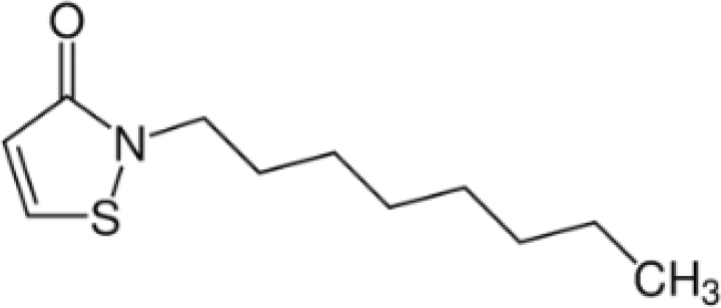	Isothioazolinone (ITs)	Interruption of oxygen consumption, enzymatic inhibition, cell compounds oxidation	26,530‐20‐1	^35^, ^51^
Iodopropynyl butylcarbamate (IPBC)	Biotin R	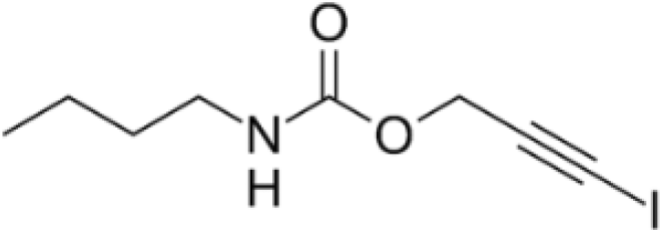	Carbamate	It affects cellular membrane permeability in fungi Highly toxic to cold‐water fish and warm‐water fish, aquatic invertebrates, estuarine/marine fish, and estuarine/marine invertebrates	55,406–53‐6	^52^
N‐(Dichlorofluoromethylthio)‐N′,N′‐dimethyl‐N‐phenylsulfamide (dichlofluanid)	Lichenicida 264	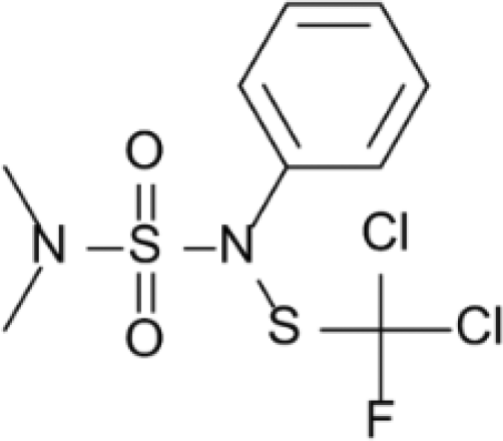	Sulfamides	Reaction with nucleophilic groups inside the cell like SH groups of enzymes leading to disulphides	1085‐98‐9	^53^
Dimethyl sulphoxide (DMSO)	DMSO‐based gels	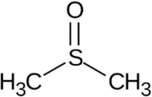	Sulphoxides	Disintegration of the membrane bilayer at high concentrations	67‐68‐5	^44^
Cholinium@halide (Halide = Br or I) and Cholinium@DBS (DBS = dodecylbenzensulphonate)	Ionic Liquids (ILs)	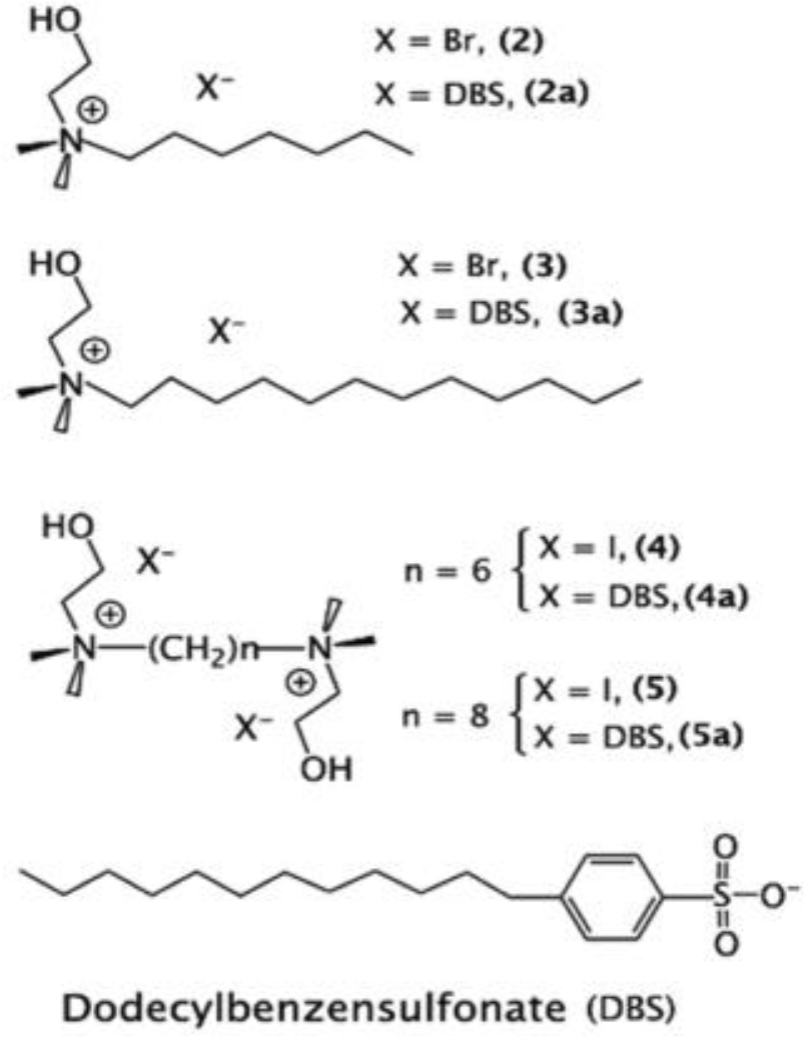	ILs	Necrosis and apoptosis induced by cytotoxicity in eukaryotes. Mechanisms poorly investigated in bacteria	Not Found	^35^
TiO_2_ nanoparticles + silver	Nanoparticles (NPs)	Not available	Nanocomposites	ROS production, coenzyme A degradation, cell wall and membrane disruption	Not Found	
Carvacrol	Origanum, common thyme, wild thyme EOs	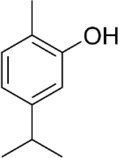	Monoterpenoid phenol	Interaction with cellular membrane leading to its disintegration; ATP depletion; ATPase inhibition; increasing of the sensitivity to antibiotics and toxic compounds	499‐75‐2	^34^, ^54^, ^55^, ^57^
Thymol		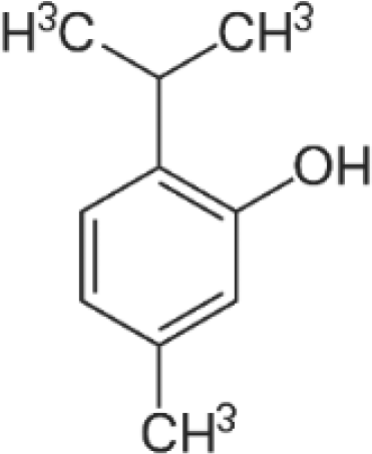			89‐83‐8	
Estragole	Fennel, basil, anise, tarragon EOs	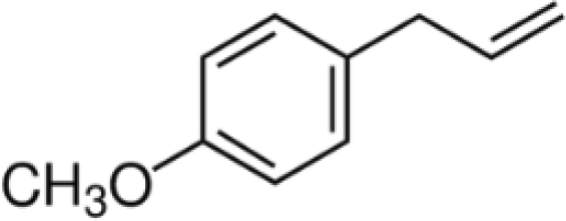	Phenylpropene	Both estragole and anethole penetrate the outer membrane in *E. coli* ^54^ Estragole is suspected human carcinogenic^56^	140‐67‐0	
Anethole		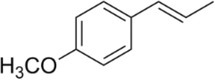	Phenylpropanoid		4180‐23‐8 *E* isomer 104‐46‐1 Unspecified stereochemistry (most commonly used CAS, implicitly *E*) 25,679‐28‐1 *Z* isomer	
Usnic acid	Lichen secondary metabolites	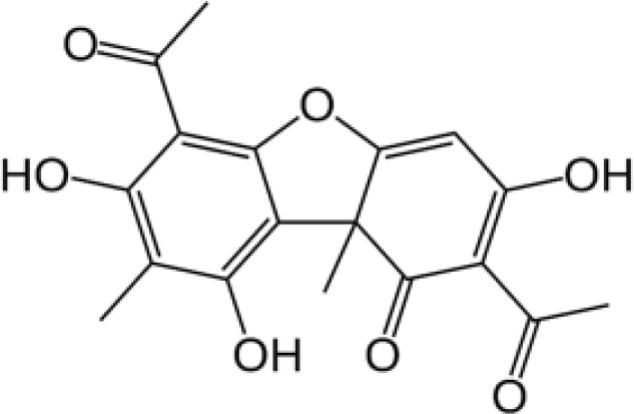	Dibenzofuran	Unknown; may be interference with RNA synthesis and DNA impairment (*B. subtilis*, *S. aureus*)	125‐46‐2	^58^
Norstictic acid		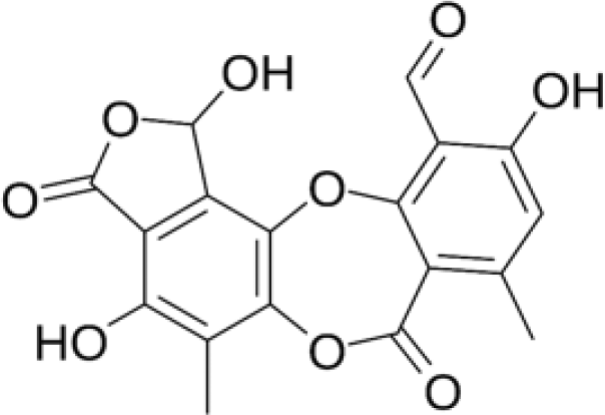	Depsidone	Unknown	571‐67‐5	
Parietin		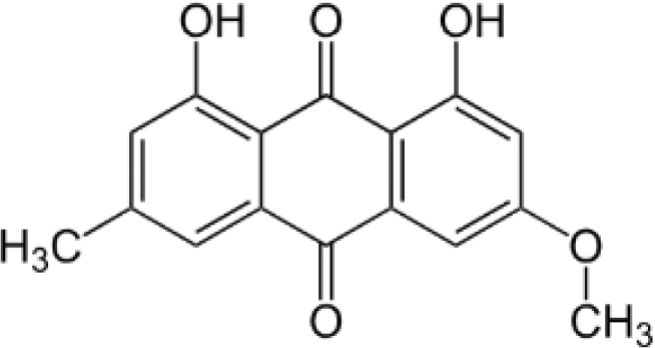	Antrhaquinone	Unknown	521‐61‐9	

Benzalkonium chloride (90%; CTS, Italy) and Preventol RI 50 (benzalkonium chloride 50%; Bresciani srl, Italy) contain benzalkonium chloride as a single active substance. This and other quaternary ammonium salts are among the most used disinfectants and antiseptics.^59^ Its antimicrobial activity is due to the positive charge that disrupts the cell membrane of the target organism even at very low concentrations (0.12%–0.13%).^48^

Lichenicida 264 (Bresciani srl, Milano, Italy) contains dichlofluanid, belonging to the N‐haloalkylthio compounds (Table 2). The biocidal activity is based on the ability of the N‐S bond to open and react with nucleophilic entities within the cell, such as SH groups of enzymes, leading to disulphide.^53^

Biotin T (CTS srl, Altavilla Vicentina, Italy) contains two active molecules (Table 2): dodecyldimethylammonium chloride (DDAC), a quaternary ammonium biocide which disrupts cellular membranes,^49^ and 2‐octyl‐2H‐isothiazole (OIT) which inhibits growth by disrupting metabolic pathways involving dehydrogenase enzymes.^35^, ^51^ The current formulation of the product also contains formic acid.

Biotin R (CTS srl, Altavilla Vicentina, Italy) contains as active molecules 3‐iodo‐2‐propynyl butyl carbamate (IPBC) and OIT dissolved in 2‐(2‐butoxyethoxy) ethanol (Table 2). The IPBC has a carbamate structure that targets cell membrane permeability and fatty acids in fungi^52^; OIT was described above.

Only two works considered for this review used exclusively conventional biocides,^18^, ^40^ the other studies used conventional biocides for comparison with innovative biocides.^6^, ^41^, ^43^, ^44^, ^45^

In the work of Isola et al.^40^ a total of 18 fungal strains were isolated from dark spots present on two Etruscan funerary tombs (Moretti tomb and Tomb of the Blue Demon) located in the Monterozzi necropolis (Tarquinia, Italy). Fifteen of them belonged to the group of black fungi (BF) and included three strains belonging to the BY group: *Exophiala angulospora* and *Cyphellophora eucalypti*. In this study, two benzalkonium chloride‐based products were chosen for the sensitivity tests: Benzalkonium chloride (90%; CTS, Italy) and Preventol RI 50 (benzalkonium chloride 50%; Bresciani srl, Italy). The sensitivity to biocides was tested using the agar diffusion test by inoculating 300 μL of an 8 ± 0.2 mg/mL (dry weight) fungal suspension into Potato Dextrose Agar by inclusion. Each biocide was applied at different concentrations of 1%, 1.5%, 2%, and 2.5% on sterile cellulose disks (6 mm diameter). Both products showed antimicrobial action against the BYs, greater at higher concentrations, even if Preventol RI 50 produced thin total inhibition halos surrounded by partial inhibition halos at all the tested concentrations.

In a more recent work of Isola et al.,^18^ 18 strains of black fungi isolated from some Carrara marble funerary monuments were tested to evaluate their sensitivity to four selected biocides. Sixteen out of 18 strains belonged to the groups of BMF and BY (genera *Aureobasidium*, *Coniosporium*, *Exophiala*, *Knufia*, *Neodevriesia*, *Neophaeotheca*, *Saccotheciaceae*, *Salinomyces*, *Saxophila*, *Vermiconidia*).

Two biocides employed contained quaternary ammonium compounds, that is, Benzalkonium Chloride and Preventol RI50; the other two were Lichenicida 264 and Biotin R. The sensitiveness to biocides was measured using the agar diffusion test as described in,^40^ Malt Agar as the nutrient medium, and the following concentrations: Benzalkonium Chloride and Preventol RI 50 0.5%, 1%, 1.5%, 2%, and 3%; Lichenicida 264 0.25%, 0.5%, and 1%; Biotin R 0.5%, 1%, 2%, 3%, 4%, and 5%. Most strains showed a low sensitiveness to Benzalkonium Chloride and Preventol RI 50, while Biotin R seemed to be more effective. *Exophiala bonariae* showed a higher tolerance when compared with other fungi.

### 2.2 Innovative biocides

In this review, innovative biocides refer to commercial or noncommercial chemical formulations that have been tested as new treatments to control microbial growth on heritage stone. The innovative biocides used against BMF as more suitable substitutes for traditional biocides are ionic liquids, dimethyl sulfoxide, and nanoparticles. Their main characteristics (chemical composition and mechanism of action) are described below and synthetically in Table 2.

Ionic Liquids, a candidate green alternative to classic solvents, are a class of materials discovered in the 18th century and defined as low melting point organic salts that possess unique properties like high ionic conductivity, high thermal stability, low vapor pressure, the ability to solvate a wide range of compounds,^60^ and recyclability. Ionic liquids have antifouling properties and, like other antifouling coatings, would be able to remove or prevent biofouling by microorganisms on wet surfaces.^61^ The perspectives of ionic liquids as an innovative antimicrobial approach for stone‐built CH have been reviewed in Lo Schiavo et al. (2020).^37^

Dimethyl sulfoxide (DMSO) is a polar aprotic solvent widely used for various pharmacological agents at concentrations of 0.05%–1.5% (v/v) with different applications in biology and medicine.^61^ It is generally considered to have low toxicity with no observable toxic effects to human cells at a 0.1% final concentration,^62^ although large‐scale deregulations of cardiac microRNAs and smaller, but still extensive, effects on hepatic microtissues were reported.^47^ Depending on its concentration, it can influence membrane stability, eventually leading to membrane disintegration at higher concentrations, by increasing membrane fluidity.^44^

Nanoparticles (NPs) are small particles with dimensions in the nanoscale range and can be categorized into various classes based on their composition and dimensions. They display a peculiar reactivity with organisms and are appealing to use both for organic and inorganic materials.^47^ NPs can be associated with other materials, giving multiphase solid materials in the nano dimension or nanocomposites. Some NPs or their nanocomposites composed of silver (Ag), copper (Cu), titanium dioxide (TiO_2_), or zinc oxide (ZnO) display interesting biocidal features.^47^ They were applied on stone in preventing biological development and yielded good results also as a preservative treatment against recolonization after conservation treatment.^63^, ^64^, ^65^

Multiple mechanisms of action are associated with NPs and nanocomposites, including disruption of the cell wall and the plasmatic membrane, inhibition of protein synthesis and DNA replication, and enhanced oxidation of cell components and compounds.^47^ These multiple, synergistic mechanisms of cytotoxic activity reduce the likelihood that the microorganisms develop resistance against the nanocompounds.^66^ Nanoparticles, in particular TiO_2_‐based NPs, are considered among the most promising treatments, although some concerns about human health and environmental risk have been raised.^36^ The pros and cons of using nanocomposites are reported in a recent review.^67^

Only one work considered for this review used exclusively an innovative treatment alone^39^; the other studies used innovative biocides together with conventional biocides for comparison^44^, ^45^ or a cleaning step with a conventional biocide in a stone treatment.^43^

In the work of De Leo et al.,^39^ the antifouling properties of Surface Active Ionic Liquids (SAILs) compounds with biocidal activity were evaluated as an alternative to conventional biocides. Their antimicrobial activity derives from their amphiphilic structure that allows their insertion inside the phospholipid bilayer of the plasma membrane leading to cellular damage and at last to cell death.^68^ Eight different newly synthesized cholinium‐based ILs were tested to evaluate their antimicrobial activity. Specifically, four of them were cholinium@halide ILs (halide = Br or I) while the other four were cholinium@DBS ILs (DBS = dodecylbenzensulphonate). ILs have been tested against *Aureobasidium* sp. strain MC 875 using the Agar disc diffusion test. Compounds were diluted in water or ethanol at 75 or 150 μM/mL and applied on 6 mm diameter sterile cotton disks put on Malt Extract Agar plates where a 1.0 × 10^6^ cell/mL suspension of *Aureobasidium* was previously spread. *Aureobasidium* was observed to be susceptible to six out of eight ILs already after 4 days of incubation, particularly the two ILs N‐(2‐hydroxyethyl)‐N,N‐dimethyl‐1‐dodecanaminium bromide and N‐(2‐hydroxyethyl)‐N,N‐dimethyl‐1‐dodecanaminium dodecylbenzenesulfonate that showed the best biocidal activity. Then these six ILs were tested on stone using four sterilized samples of Carrara marble and tufa (5 × 5 × 1.5 cm), on which squares of 2.5 × 2.5 cm were engraved to obtain four sectors. The samples were treated with a double layer of a commercial consolidant (NanoEstel) and kept at 20°C for 3–4 days to ensure the polymerization of the product on the stone surfaces. After that, the samples were sterilized again and, finally, ILs were applied with a sterile sponge (as for the consolidant) at the concentrations of 4.69 μmol/mL (four samples) and 2.34 μmol/mL (two samples). A stabilized suspension of a mix of microorganisms including bacteria, an alga, and the fungi *Cladosporium* sp. and *Aureobasidium* sp. was applied on the treated and untreated stones to simulate natural colonization. The samples were kept for 3 months in glass containers and incubated under light conditions (1200 lux) at room temperature (25°C) and constant humidity. On tufa samples, after 90 days, four ILs caused the complete drying of the biofilm and the disappearance of the algae. Microscopical analyses showed that two of the most effective ILs caused a significant decrease in microbial cells. However, black yeasts were still present (confirmed by cultivation). On the other hand, the other two ILs (N‐(2‐hydroxyethyl)‐*N*,*N*‐dimethyl‐1‐dodecanaminium bromide and N‐(2‐hydroxyethyl)‐*N*,*N*‐dimethyl‐1‐dodecanaminium dodecylbenzenesulfonate) worked very well also against fungi. On marble samples, after 90 days of incubation, the last two ILs led to the biofilm drying and yellowing. Un‐inoculated tufa and marble samples (treated and untreated) were used to assess any spontaneous colonization and the same ILs were able to prevent and slow down spontaneous colonization. Hyphomycetes with elongated and dark‐colored conidia belonging to *Cladosporium* were observed under the stereomicroscope, while no indications were reported for BMF/BY.

Three works are centered on the use of NPs and DMSO‐based gels. Ruffolo and collaborators^43^ conducted a medium‐term experiment using TiO_2_ nanoparticles mixed with Ag on monumental stones of the archeological site of “Villa dei Papiri” (south‐east wall) located within the Ercolano site (Naples, Italy). Before any nanoparticle treatment, the wall was treated with a solution of 5% Biotin R (CTS, Italy) as previous cleaning step, using a brush for three times (at 15 days of time interval). Two days after the last treatment with Biotin R, the surface was washed with ddH_2_O. Then, after 1 month, nano‐biocides were applied on the stone for the treatment. Formulations used for the treatment include pure TiO_2_ (anatase 25 nm size), TiO_2_ mixed with silver nanoparticles (100 nm size; TiO2/Ag ratio 100/1) dispersed in aqueous dispersion of nanosilica. Blends containing different concentrations of nanosilica were used (B5% and B10% wt) while TiO_2_ concentration was always 1% wt. The application was performed with a brush on the stone surface in the amount of 400 mL/m^2^. Twenty‐two samples were collected using a nondestructive method (adhesive tape) from the site before any treatment at different heights (samples ERB 1–8), after Biotin R treatment (samples ERBD 1–5), after TiO_2_ treatment (samples ERBN 1–3, 5–8), and from the surfaces subsequently treated with the only binder n‐SiO_2_ (control; sample ERBN 4 and 9). Fungi were cultivated from adhesive tapes on the medium Dicloran Rose Bengal Chloramphenicol (DRBC). The results obtained from cultural analysis prior to treatments indicated the presence of several BF, including the BMF *Cyphellophora* sp. and *Devriesia* sp. and the BY *Aureobasidium pullulans*. After the treatment with Biotin R, no fungal strain was obtained from cultivation except for one sample (ERBD3) from which *Alternaria alternata* was isolated. The occasional presence of fungal colonies of different groups was observed from all the sites treated with TiO_2_ based products 4 months after, however, black fungi were isolated only from the ERBN4 site (control).

In the work of Toreno and collaborators,^44^ a low impact and inexpensive procedure for the prevention and control of biodeteriogens using dimethyl sulfoxide (DMSO)‐based gels was investigated. DMSO‐based gels dissolve the EPS of the biofilm matrix, facilitating their removal.^45^ The preliminary interaction between the gel and the stone was performed on an ordinary, smooth but not glossy, uncolonized Carrara marble (20 × 20 cm). The tests did not significantly modify the physical parameters of the probe, as well as the rugosimetric and colorimetric properties. Right after, field tests were performed on two cemetery marble monuments located in the Monumental Cemetery of Cagliari and diffusely colonized: specifically, a funerary cross and an angel (Frau‐Carta), and a marble fragment. The cross was used for the preliminary tests to verify the effectiveness and the impact of the gel on the colonized surface; the marble fragment was used to compare the effectiveness of the gel with different biocide‐based methods, while the angel head was used to verify the long‐term efficacy of the gel. For the characterization of microbial biofilms before any treatment, nondestructive methods for both microscopic and cultivation purposes were employed. Cultivation tests were repeated on the marble fragment 1 month after and from the angel head 1 year after the treatment, to verify short and medium‐ to long‐term recolonization. Two gels were prepared: Gel 1, using 100 mL of DMSO, 10 mL of Ethomeen C25 (8.41% w/w), and 3 g of Carbopol 934 (2.44% w/w); Gel 2, with the same composition as Gel 1 where DMSO was replaced with distilled water as control. 3% Preventol RI50, 2% Lichenicida 264, and 5% Biotin R were used for comparative studies. Before the treatments, microscopic analysis confirmed the presence of BMF on the funerary cross surface and the marble fragment, while on the angel head the presence of BMF species like *Knufia mediterranea*, *K. marmoricola*, and *Neodevriesia sardiniae* was confirmed by a previous study.^27^ Black fungi growing as microcolonies were present on the marble specimen. The results obtained on the funerary cross indicated that Gel 1 had a cleaning effect already after the first application, while after six applications, the cleaning effect was deeper (confirmed also by SEM analysis). The comparative study performed on the marble fragment was evaluated visually and via colorimetric and microscopic analysis and showed that the cleanest surface was obtained using Gel 1. One month after the treatments, cultivation from the marble fragment gave no viable colonies from the samples taken where the commercial biocides and Gel 1 were applied; on the contrary, for the negative control Gel 2. However, the areas treated with commercial biocides still presented colored spots. Gel 1 gave excellent cleaning results after two applications on the angel head; furthermore, no colonies were obtained from the cultivation of samples taken 1 year after the treatments.

The same authors^45^ investigated the recolonization dynamics after 9 years, following the removal of biopatinas by using the two DMSO‐based gels as described above and the commercial product Biotin T on three marble monuments located in the Monumental Cemetery of Cagliari (Italy). This is one of the few works available in literature about long‐term monitoring of recolonization after an antimicrobial treatment. The monuments (Frau‐Carta (FC),^44^ Giuseppina Ara Ciarella (AC), and Francesca Warzee (FW)) were chosen for the study. FW and FC were treated using the DMSO‐based gel, while AC was entirely treated with Biotin T due to restoration purposes. Microbial recolonization of stone was investigated by focusing on RIF detection, diversity, and distribution, since their remarkable tolerance to a wide range of environmental conditions and chemicals. The three monuments were sampled both before and after treatment in summer 2015, winter 2018, and summer 2022. Samples were taken with sterile swabs and adhesive tapes. Swabs were washed with sterile water and suspensions were plated onto DRBC; the growing RIF colonies were counted and then isolated onto MEA and phylogenetically placed by rDNA ITS analysis. The treatment performed with DMSO‐based gels on FC and FW seemed to be more effective against black fungi compared to the Biotin T‐based treatment on AC. Two years after the DMSO treatments, the viable count of black fungi showed a drastic reduction, a little less pronounced for the Biotin T treatment. After 5 and 9 years, the BF viable count slowly increased for DMSO treatments, keeping lower than the initial count. Five years after the treatment with Biotin T, the number of BF colonies was not significantly different from that before the treatment, and after 9 years, the BF count was higher than the initial one, while the number of other fungi decreased, leading to a higher ratio BF/total fungi. Moreover, the Biotin T treatment produced an increase also in BF biodiversity and resistant species. After the treatments on AC, some species isolated before the treatment disappeared (*V. calcicola*, *A. pullulans*), *K. petricola* was reisolated while species like *K. marmoricola* and *K. mediterranea* appeared for the first time; moreover, new not identified *Dothideomycetes* sp. and *Eurothiomycetes* sp. were found.

## 3 BIOCIDES FROM NATURAL SOURCE

Natural biocides derive from various organisms and are considered safer compared to traditional ones.^38^ They have been extensively considered against the biodeterioration of stone cultural heritage and, among them, essential oils (EOs) appear to be highly preferred due to their well‐recognized antimicrobial activity investigated in several fields.^38^

Natural biocides tested against BF are lichen secondary metabolites and EOs. In two studies^6^, ^41^ they were tested together with conventional biocides for comparison.

Lichen secondary metabolites (LSM) are a group of extracellular metabolites^69^ exclusively synthesized by the mycobiont.^70^ These molecules are produced by the lichen to gain protection in negative conditions (physical and biological).^71^ LSM chemical properties and biological potential have been extensively treated by Goga et al.^69^ LSM could be employed for pharmaceutical, biological, and ecological scopes, thanks to their antibiotic, antiviral, and antiproliferative properties.^71^ As also evidenced by Gazzano and collaborators,^41^ their role against rock‐dwelling organisms has been neglected.

EOs are complex mixtures of substances, mainly aromatic compounds, produced by plants as secondary metabolites with high chemical variability and broad‐spectrum activities, including antimicrobial properties, which have been employed for centuries in folk medicine^72^, ^73^ They are readily available commercially and are commonly used in pharmacology, aromatherapy, food, agriculture, and cosmetics.^74^ Some EOs are very effective against bacteria, algae, and fungi at low concentrations.^35^ Among the effects produced on microbial cells leading to the inhibition of its growth, there are inducing the deterioration of the cytoplasmic membrane, regulating intermediary metabolism, activating or inhibiting enzymatic reactions, or affecting enzyme synthesis.^72^ Some of these effects can be attributable to single components of EOs, such as carvacrol and thymol, able to permeabilize and depolarize the cytoplasmic membrane of *Escherichia coli*; however, their action mechanisms remain poorly understood, also due to their high chemical variability.^34^

Gazzano and collaborators^41^ investigated the biocidal potential of three lichen secondary metabolites (LSM): usnic acid (UA), parietin (P), and norstictic acid (NA) to control biopatinas on cultural heritage stones, including white Carrara marble. Prior to any test on stone, LSM were tested for their safety toward marble specimens; the application of the LSM did not produce any appreciable chromatic alterations of marble. Nevertheless, this should be better confirmed by simulated aging on the stone surface, as commented by the authors. All the three LSM were solubilized in a 10/90 v/v mixture of acetone/water. The final concentrations of the three LSM were 0.05 mM for NA and P, and 0.02 mM for UA. LSM were tested against different BMF, specifically *Coniosporium uncinatum*, *C. apollinis*, *C. perforans*, and *Phaeococcomyces chersonesos*. Water and 10% acetone served as negative control, while benzalkonium chloride approximately 0.03 M served as positive control. The tests were conducted on agar plates, where all the compounds (50 μL) were spread around each previously grown colony at a distance of about 0.7 mm. The plates were incubated at 15°C for 30 days, after which growth measurements based on colony area were carried out by image analysis. The results indicated a strong inhibitory effect of the LSM on all the four BMF, statistically the same as benzalkonium chloride. The effect was stronger on the *Coniosporium* species rather than *Phaeococcomyces* sp.

Mironescu and Georgescu^42^ investigated the efficacy of six volatile oils extracted from five plants (*Pinus sylvestris*, *Juniperus communis*, *Abies alba*, *Thymus serpyllum*, *Thymus vulgaris*, and *Foeniculum vulgare*) on different fungi, including the BY *Aureobasidium* sp. isolated from two old buildings. The test was conducted using fungal spore suspensions prepared from previous cultures and inoculated on Czapek‐Dox medium. Approximately 8 μL of essential oils were applied to Whatmann discs of 5 mm diameter and placed at the center of the Petri dish. The inhibition efficacy was evaluated with the agar diffusion test measuring the diameter of the inhibition zone. The results showed a great inhibition activity of *T. vulgaris* (100%—full inhibition), *T. serpyllum* (93%), and *F. vulgare* (95%) EOs against *Aureobasidium* in comparison to untreated colonies, maybe because of their alcoholic component.

In 2021, Santo et al.^26^ demonstrated that the darkening of the external white marble covering the Cathedral of Santa Maria del Fiore (SMFC; Florence, Italy) was due to the growth of black fungi and dark cyanobacteria. The presence of RIF was demonstrated by microscopic analysis in those darkened areas, and some BFM/BY strains were cultivated from marble together with filamentous black fungi on MEA, although the conditions adopted for cultivation were not suitable for RIF growth. The same authors demonstrated that the essential oils (EOs) extracted from *Origanum vulgare* L. (EO‐Origanum) and *Thymus vulgaris* L. (EO‐Thymus) were effective in breaking down the microbial load of the mold and bacterial communities cultivable from the darkened marble at the very low concentration of 0.25% (v/v) by plating SMFC marble suspensions on nutrient media with and without EOs.^34^ Following these results in lab conditions, an in situ treatment with the two EOs at the working concentration of 2% (v/v) was carried out on the marble surface of the same darkened areas, where their effectiveness was compared with that of a 2% solution of Biotin T.^34^ Results showed that EOs and Biotin T were both efficient in significantly reducing microbial viability and vitality (measured as viable titer and ATP content, respectively). These results led the authors to investigate the RIF community inhabiting the SMFC marble and to extend the research on EOs efficacy in a subsequent work.^6^ The presence of a high diversity community of RIF in the darkened marble areas was demonstrated. Twenty‐four strains were isolated by cultivation on DRBC medium and phylogenetically characterized by a multilocus analysis. After that, eight strains (*Knufia* sp., *Lithohypha* sp., *Vermiconia* sp., *Coniosporium* sp., *Dothideomycetes* sp.) out of 24 were chosen to assay their sensitivity to the products already used in situ by a plate test: EO‐Thymus and EO‐Origanum (Erbamea, s.r.l., Italy), and Biotin T. Each 25% (v/v) stock solution (EOs dissolved in DMSO, and Biotin T in water) was incorporated into MEA medium and used in a range of very low concentrations (0.0025–0.025%v/v) with respect to those used for stone treatment, where the highest was that (0.025%) previously demonstrated as effective against the cultivable community of the SMFC marble.^34^ Variations of colony diameters were measured for each strain in triplicate after 28 days of incubation at 20°C, and the average growth obtained on plates with biocides was compared with the control (growth on MEA with DMSO) and data were statistically analyzed. In the considered range, the tolerance to biocides was strain‐specific and concentration‐dependent. Surprisingly, EOs were more effective than Biotin T at the highest concentration tested (0.025%), with EO‐Origanum completely inhibiting all the tested strains, while all the strains could still grow in the presence of Biotin T. At the lowest concentrations of 0.0025% and 0.005%, EO‐Origanum and Biotin T had comparable inhibitory effects.

## 4 PHYSICAL METHODS

The only work available reporting a physical method for the control of BMF was carried out by Cuzman et al.^75^ The microwave heating is a promising application to control biodeteriogens on both organic and inorganic materials of works of art^47^; nevertheless, the microwave treatment may present some drawbacks if not correctly performed, such as the presence of highly heated areas (e.g., hot spots) or areas with poor radiation due to specific shapes.^76^ To overcome this issue, Pierdicca and colleagues^76^ proposed a mathematical model allowing predicting and monitoring tasks about the heating process applied to cultural heritage objects.

The work of Cuzman et al.^75^ served as a preliminary study on the application of microwave for the control of BF growing on stone monuments. The study was carried out on different fungal strains previously isolated from three monuments, including the BMF *Sarcinomyces* sp. (reclassified as *Knufia* sp.). The test was conducted on single colonies grown on MEA plates for 10 days, irradiating different doses of microwave directly onto the plates. A fully portable microwave heating system was used with a 2.45GHz generator with an output power of up to 1 kW. The plates were always maintained at a fixed distance of 3 mm from the applicator aperture to uniformly heat the colonies. Different temperatures and times of exposition were tested: 55°C for 3, 6, and 9 min and 65°C for 3 min. The results showed that the 55°C for 3 min treatment led to little damages to the mycelium, the 55°C for 6 min treatment led to severe damages to the mycelium, but alive cells were still present, while the 55°C for 9 min and 65°C for 3 min treatments led to the complete killing of the colonies. The viability of cells was evaluated by optical microscopy using the fluorescent probes fluorescein isothiocyanate and propidium iodide and by inoculating a small fragment of the mycelium on new MEA.

## 5 DISCUSSION AND PERSPECTIVES

BMF and BY are widely known to be extremely aggressive agents toward artwork of heritage stone.^4^, ^29^, ^77^, ^78^ Despite this, very few works are available with RIF as a specific target for antimicrobial tests, mostly in laboratory conditions (Table 1). So, as a first need for future work, we underscore the necessity of a more extended and in‐depth investigation on RIF sensitivity to chemical and physical treatments, especially in situ. It is well known that life and growth conditions of microorganisms in the environment are quite different when compared with those in laboratory settings. BMF and BY exhibit additional characteristics that complicate their treatment on stone surfaces, including their slow growth rates, endolithic lifestyle,^77^ and resilience to various environmental stresses.^79^ Studying their responses to antimicrobial treatments on stone can not only contribute to evaluating their sensitivity to cleaning methods but also provide valuable insights into the genetic resistance mechanisms of RIF.^80^ Despite the limited number of works found, most of them use different innovative biocides and natural products, comparing their effectiveness with that of traditional biocides and dealing with very different experiments depending also on the objective. In general, to control microorganisms responsible for the biodeterioration of monuments, the purposes of the work can be different and basically refer to two kinds of approaches. One kind of approach involves the study of a single case to find the most effective treatments against microorganisms deteriorating a particular monument and is mainly aimed at giving concrete and immediate answers to the restorers. This is the case of the works of Isola et al.^40^ and Isola et al.,^18^ which are the only two works that tested exclusively traditional biocides (Table 1). The second approach focuses on the development of new treatments that balance efficacy with eco‐sustainability. This is evident in the various case studies reviewed here, where innovative chemical and physical solutions have been tested to control RIF growth. Among these, some are studies on single or more RIF strains, mainly isolated from the monuments under study, carried out in laboratory conditions, and some others in the field (Table 1), showing a mixed picture of the state of the art. Moreover, one or more innovative chemical products have been used, while only one work used a physical method based on microwave, in laboratory conditions.

Other than the differences in the application procedures, partly due to the differences in the products used, another observation gained from this review is the heterogeneity of methods employed to evaluate antimicrobial treatment effectiveness and the infrequent application of statistical analysis to substantiate the findings (Table 1). The standardization of the test conditions would be particularly important for in‐field studies, where a multidisciplinary approach, spanning from cultivation to microscopy, is often used without any agreement on methodologies. The fact that all the works discussed in this review are separate case studies makes it difficult to define standard methods for cleaning black fungi from stone. When each study focuses on a unique case, often in varying environmental conditions and involving different types of stone and chemicals, it becomes even more challenging to create universally applicable guidelines.

As recently highlighted by Urzì,^81^ international guidelines could provide a valuable framework, allowing restorers, conservators, and scientists to compare methodologies and results obtained in different climates and conditions. Stone artworks affected by RIF are often exposed to external environments, making climate a crucial factor in treatment outcomes. As a first problem, there is no universally accepted language for describing material alterations and diagnosis of the problem, which should be the first step toward standardization for guidelines. The European Commission has structured working groups focused on cultural heritage, beginning with common definitions, much like in the medical field.^81^ A second critical aspect is the development of standardized sampling procedures. In this context, the UNI EN 16085:2012 “Conservation of Cultural Property – Methodology for Sampling Materials Constituting Cultural Property – General Rules” offers guidelines ranging from noninvasive to invasive techniques.^81^ A third important point involves the evaluation of treatments, as outlined in the EN 17138:2018 “Conservation of Cultural Heritage for Cleaning Porous Inorganic Materials.”

While the above considerations are broadly applicable to addressing stone biodeterioration, mitigating the presence of RIF on stone monuments presents unique challenges. These difficulties arise not only from their endolithic nature, stress resistance, and survival strategies, as previously mentioned, but also from challenges in identification due to limited studies and a lack of genetic sequences in databases. Moreover, their high genetic variability and strain diversity further complicate treatment efforts. Another significant ecological factor is their association with other organisms, such as lichens, which can exacerbate the damage to heritage stone and hinder effective containment. Both culture‐dependent and culture‐independent approaches demonstrate that black fungi are frequently associated with lichens co‐occurring with other fungi within lichen thalli, and some black fungi have a transient capacity to optionally develop lichen‐like associations with algae or cyanobacteria, which can also be observed in natural and urban surfaces of rocks and concrete.^82^ These associations complicate conservation and cleaning efforts, as the strong attachment of lichens to stone surfaces, combined with the melanin produced by BF, could make removal more difficult. Research aimed at uncovering the mechanisms by which black fungi and their microbial partners gain nutrients, adapt to harsh environmental conditions, and affect stone deterioration will be crucial for developing more effective conservation strategies. This area remains a promising and important avenue for future investigation.

Therefore, considering all the aforementioned factors, at the current stage of knowledge and in our view, it is not feasible to propose any Standard Operating Procedure for cleaning stone damaged by black fungi, but it is reasonable to set this goal. Again, it is useful to consider the purpose of the intervention on stone. When the aim is the mitigation of black fungi on a specific monument, a more realistic approach is the evaluation case by case of the efficiency of biocides on selected strains isolated from the monument to be able to give the right information to the restorers who then have to apply the products. On the other hand, in the case of experiments aiming to test newly promising treatments with biocidal and/or antifouling properties, the use of reference strains and standard methods is very advisable, especially if the product should be commercialized.

Synthesizing these critiques, for RIF, it would be beneficial to select certain reference genera from the most known fungi commonly occurring on stone and standardize test conditions, both in the laboratory and in situ, for evaluating their sensitivity. The double‐aim objective would be to determine the effectiveness of treatments and to explore the mechanisms of sensitivity and resistance, including the genetic basis of biocide resistance. In this regard, the genus *Knufia* lends itself well as a possible reference. Among *Knufia* species, *K. petricola* is widespread on cultural stone where it acts as a damaging agent,^80^ an advanced genetic toolkit to manipulate this fungus is available and has recently been used to explore its biology,^80^, ^83^, ^84^ and the genomes of more strains have been sequenced.^85^ *K. petricola* belongs to an ancestral lineage of Chaetotryales (Ascomycetes) and could be pivotal to understanding the evolution of Ascomycetes lifestyles. The strain A95 of *K. petricola* is a well‐suited model fungus to study organism–organism and organism–material interactions in RIF, which are of huge importance in the field of conservation of cultural heritage.^84^ Moreover, proteomics has been carried out on the RIF *K. chersonesos*.^86^ These pioneering studies paved the way for the genetic and molecular investigation of BMF interactions with materials like stone, other organisms, environmental factors, and biocides. In fact, a genetic model in combination with genome sequencing is essential to perform functional genomics or proteomics/metabolomic studies for the identification of genes and functions to investigate the biological basis of RIF polyextremotolerant resistance and high resilience. For example, the mechanisms of biocide resistance in the *Knufia* resistant strains that appeared on stone after treatment with Biotin T in the work of Toreno et al.^45^ could be better investigated on strains with known genomes. However, to achieve this topic, it would be necessary to have access to a certified collection and possibly to test more characterized strains of the selected species of *Knufia* known to be stone decay microorganisms, since the response to selected biocides should be very different due to the genetic variability.

Concerning the type of treatment to develop to control RIF growth, our suggestion is a future stronger support on innovative or natural biocides also in accordance with the European Green Deal (EGD,^87^) which has as its main goal to create an environment that is as “toxic free” as possible and provides protection for everyone from toxic substances. The search for alternative molecules/treatments in CH would fit particularly with point 2 of the EGD, which specifies the intention to minimize and replace as much as possible the presence of substances of concern in all products, unless a given substance is ascertained to be essential to society.

Although biocides are toxic and some concerns are legitimate, the risk they pose depends upon the circumstances of exposure and on the toxicity level.^74^ For example, IPBC contained in Biotin T and R has been approved by EU for its use as a “working or cutting fluid” preservative according to the BPR (EU) 528/2012 (https://echa.europa.eu/home). Despite of this, studies on IPBC indicate that it is highly toxic to cold‐water fish and warm‐water fish, aquatic invertebrates, estuarine/marine fish, and estuarine/marine invertebrates.^52^ Alternative biocidal methods for stone conservation should be equally effective as traditional ones but with lower acute and chronic toxicity. DMSO‐based gels and EOs, according to the few information about their use on RIF, seem to be as effective as the traditional biocides.^6^, ^34^, ^42^, ^44^, ^45^ To date, innovative and natural biocides are not known to cause evolution of resistance as in the case of compounds like benzalkonium chloride which could cause resistance when used at sub‐inhibitory doses.^88^ On the other hand, also alternative biocides can be dangerous (e.g., estragole has been demonstrated to be carcinogenic in mice and suspected carcinogenic in humans; Table 2). Due to their limited applications, the safety of natural biocides towards human health, the environment and heritage stone should be better investigated. The concerns and open issues in research in alternative methods, such as natural substances, to traditional ones to contrast biodeterioration of stone have been discussed in Pinna.^74^

At the same time, further research is needed to test the effect of innovative treatments on bioreceptivity and recolonization of stone in the long term. In this regard, the study of Toreno et al.^45^ is the only one available on the long‐term resilience of BMF on stone after cleaning treatments, evaluated by cultivation. As a general rule, according to Sterflinger and Piñar,^89^ the application of any chemicals, either innovative or conventional, must be carried out only after rigorous tests adapted to the characteristics of the heritage object and its environment.

We stress the importance of using also ‘multi‐omics’ approaches to better evaluate the effects of cleaning treatments and monitor microbial consortia on CH.^90^, ^91^ By analyzing environmental DNA samples from rock surfaces, these methods can identify and quantify RIF species present, track changes over time, assess shifts in community composition before and after biocide treatments, and evaluate the long‐term efficacy of biocides and the potential development of resistance in the RIF communities. A combined cultivation‐omics approach could offer a powerful tool to monitor RIF dynamics and processes, providing helpful information to choose suitable and targeted anti‐RIF treatments.

## CONFLICT OF INTEREST STATEMENT

All authors declare that they have no conflicts of interest.
